# Nisin Z Production by Wild Strains of *Lactococcus lactis* Isolated from Brazilian (Italian Type) Fermented Sausage

**DOI:** 10.1155/2020/9309628

**Published:** 2020-04-14

**Authors:** Margarete Alice Fontes Saraiva, Dagim Jirata Birri, Dag Anders Brede, Maria Cristina Baracat-Pereira, Marisa Vieira de Queiroz, Ingolf F Nes, Célia Alencar de Moraes

**Affiliations:** ^1^Departamento de Microbiologia, Universidade Federal de Viçosa, Viçosa, Brazil; ^2^Department of Microbial, Cellular and Molecular Biology, Addis Ababa University, Addis Ababa, Ethiopia; ^3^Department of Environmental Sciences, Norwegian University of Life Sciences, Ås, Norway; ^4^Departamento de Bioquímica e Biologia Molecular, Universidade Federal de Viçosa, Viçosa, Brazil; ^5^Department of Chemistry, Biotechnology and Food Science, Norwegian University of Life Sciences, Ås, Norway

## Abstract

In this study, five bacteriocin-producing *Lactococcus lactis* strains were identified from different naturally fermented Brazilian sausages. Ion exchange and reversed-phase chromatographies were used to purify the bacteriocins from culture supernatant of the five strains. Mass spectrometry (MALDI-TOF/TOF) showed that the molecular masses of the bactericoins from *L. lactis* ID1.5, ID3.1, ID8.5, PD4.7, and PR3.1 were 3330.567 Da, 3330.514 Da, 3329.985 Da, 3329.561 Da, and 3329.591 Da, respectively. PCR product sequence analysis confirmed that the structural genes of bacteriocins produced by the five isolates are identical to the lantibiotic nisin Z. Optimal nisin Z production was achieved in tryptone and casein peptone, at pH 6.0 or 6.5. The most favorable temperatures for nisin Z production were 25°C and 30°C, and its production was better under aerobic than anaerobic condition. The type of carbon source appeared to be an important factor for nisin Z production. While sucrose was found to be the most efficient carbon source for nisin Z production by four *L. lactis* isolates, fructose was the best for one isolate. Lactose was also a good energy source for nisin Z production. Surprisingly, glucose was clearly the poorest carbon source for nisin Z production. The five isolates produced different amounts of the bacteriocin, *L. lactis* ID1.5 and ID8.5 isolates being the best nisin Z producers. DNA sequence analysis did not reveal any sequence differences in the *nisZ* and *nisF* promoter regions that could explain the differences in nisin Z production, suggesting that there should be other factors responsible for differential nisin Z production by the isolates.

## 1. Introduction

Lactic acid bacteria (LAB) constitute a diverse group of bacteria that produce lactic acid as a major end-product of hexose fermentation. They are widely used as starter cultures in the production of many fermented foods [1]. Appropriate cultures have been isolated from naturally fermented food for use in industrial production. The use of starter cultures is based on the distinctive sensory and technology qualities that they add to the fermented products. In addition to their role in food production, starter cultures that produce antimicrobial substances, such as bacteriocins, may serve to prevent food-borne diseases and to increase the shelf-life of foods by reducing/eliminating pathogens and spoilage bacteria in fermented foods, such as sausages [2, 3]. Bacteriocins are ribosomally synthesized antimicrobial peptides and proteins produced by Gram-negative and Gram-positive bacteria [4]. One group of bacteriocins is lantibiotics, which are small, heat-stable, posttranslationally modified bacteriocins [5]. The best examples of lantibiotics are the nisins, which are most commonly produced by *Lactococcus lactis* strains and include nisins A, F, H, J, Q, U, and Z [6–13].

Nisin-producing *Lactococcus lactis* is applied in fermented foods (mostly dairy products), and it is generally recognized as safe. Nisin A was the first bacteriocin approved and commercially employed as food preservative [14]. The genetic locus of nisin A consists of eleven genes (*nisABTCIPRKFEG*) organized into three operons [13]. Transcription of nisin genes is regulated by three promoters, of which the promoter preceding *nisRK* is constitutive, whereas the *nisABTCIP* and *nisFEG* promoters are controlled by the two-component regulatory system NisRK [15]. The NisRK-mediated regulatory system responds to changes in environmental factors [16].

In view of the widespread use of this bacteriocin, an important factor to consider for its application is the cost of production. It is well known that bacteriocin production in fermentation systems is influenced by many factors, such as type of carbohydrate, nitrogen source, pH, temperature, and other nutritional and physiochemical properties [11, 17–19]. The criteria for the selection of a good starter strains include also their ability to produce bacteriocins during the conditions of growth in the fermentation process.

Most nisin-producing *L. lactis* strains have been isolated from cheese, raw milk, grain, fish, and fermented vegetable [6, 10, 13, 20–22]. The most common bacteriocinogenic LAB isolated from meat and fermented meat products are *Pediococcus* and *Lactobacillus* species [23–26].

In our previous studies, bacteriocinogenic LAB were isolated during the natural fermentation of sausage (Italian type) processed in two industries located in the cities of Imbituva and Prudentópolis (Paraná State, Brazil) [27]. The purpose of this study was to identify bacteriocins produced by five LAB strains and to investigate their performance with respect to bacteriocin production in batch culture under different growth conditions.

## 2. Materials and Methods

### 2.1. Bacterial Strains and Culture Conditions

All LAB used in this study were grown at 30°C in LAPT broth [28]. Bacteriocin-producing *Lactococcus lactis* strains were isolated from naturally fermented sausage (Italian type) (21). The susceptible strain *Micrococcus luteus* ATCC 10240 was used as an indicator strain for bacteriocin activity. It was grown at 30°C in basal media containing (per liter): animal peptone (10 g), yeast extract (4 g), meat extract (8 g), NaCl (5 g), Na_2_HPO_4_ (2.5 g), and glucose (10 g).

### 2.2. Identification of Nisin-Producing Isolates

The nisin-producing isolates were identified to species level by partial 16S rRNA gene sequence analysis. Genomic DNA was isolated with Wizard Genomic DNA purification Kit (Promega, USA). The PCR was performed by using combinations of primers, fd-^5′^CCGAATTCGACAACAGAGTTTGATCCTGGCTCAG^3ʹ^ and md-^5ʹ^CCCGGGATCCAAGCTTAAGGAGGTGA-TCCAGCC^3ʹ^ [29], under the following conditions: an initial denaturation at 95°C for 2 min, followed by 30 cycles of denaturation at 95°C for 2 min, annealing at 58°C for 30 s and extension at 72°C for 2 min, and a final extension at 72°C for 5 min. PCR products were purified and sequenced, and the sequences were compared with those in the GenBank database using BLAST software provided online by National Center for Biotechnology Information (USA) to determine the closest known relatives of the partial 16S ribosomal gene sequence of the isolates.

### 2.3. Sequencing of Nisin Structural Gene and Two Promoters

Nucleotide sequencing was performed on the PCR products obtained from the amplifications of genomic DNA with primers specific to *nisA* structural gene and *nisA*, and *nisF* promoters (designed according to the nisin A regulon, GenBank: HM219853.1). The PCR thermal cycle program included an initial denaturation at 94°C for 2 min, followed by 35 cycles of denaturation at 94°C for 1 min, annealing at 40°C, at 48°C, and at 68°C for 30 s for the primers sets nqf^5ʹ^CGTTCGAAGGAACTACAAAATAAATT^3ʹ^/naqzr^5ʹ^ACAGACCAGCATTATATTTCTGC^3ʹ^, pnisAf^5ʹ^TTGAGTCTTAGACATACTTGAAC^3ʹ^/pnisAr^5ʹ^CAATGACAAGTTGCTGTTTTCA^3ʹ^, and pnisFf^5ʹ^TCCTCAAAAAGGTGGGGCAGAAGT^3ʹ^/pnisFr^5ʹ^GCCTCGATTAAGGCTCCAAGT^3ʹ^, respectively [30], and extension at 72°C for 1 min. The final extension was performed at 72°C for 7 min. The amplicons were purified and sequenced, and sequences were compared with those in the GenBank database as described above.

### 2.4. Curing of Plasmids

Plasmid curing was done in order to determine whether or not the genetic determinants for bacteriocin production in the five *L. lactis* strains are located on plasmid. *L. lactis* strains were grown in LAPT broth and 10 *μ*g mL^−1^ of ethidium bromide at 30°C for 24 h. After incubation, the same procedure was repeated several times. Cultures, which survived were diluted, plated on LAPT agar, and incubated at 30°C for 24 h. Colonies were further screened for bacteriocin production. The plasmids of the wild type and variants were checked by EZ N. A. ™Plasmid Spin Protocol (Omega, USA).

### 2.5. Bacteriocin Antimicrobial Activity Assay

Quantitative determination of the antimicrobial activity of the bacteriocins was performed by using agar well diffusion assay [31]. Preparations of the cell-free culture supernatant (boiled and neutralized) as well as purified bacteriocin were serially diluted and tested against indicator strain. One arbitrary unit (AU·mL^−1^) was defined as the reciprocal of the highest dilution that showed a zone of inhibition of at least 5 mm in diameter.

### 2.6. Bacteriocin Purification and Mass Spectrometry

Bacteriocins were purified from the culture supernatant of the five *L. lactis* strains using ion exchange and revered-phase chromatography. The bacteriocins were precipitated by using ammonium sulfate (40%), dissolved in water, and the pH was adjusted to 3.5. The preparation was then passed through SP Sepharose Fast Flow (GE Healthcare Biosciences, Uppsala) equilibrated with 10 mmol·L^−1^ acetic acid. The column was eluted with a stepwise gradient consisting of 10 mL of each 0.1, 0.3, and 1.0 mol·L^−1^ NaCl at 1 mL·min^−1^ flow rate. The fractions that showed the highest bacteriocin activity were fractionated on a reversed-phase column (Resource 15 RPC; Pharmacia Biotechnology) equilibrated with 0.1% (v/v) trifluoroacetic acid (TFA) in water, using Äkta Purifier Fast Protein Liquid Chromatography System. Elution was performed by using a linear gradient from 0 to 100% isopropanol containing 0.1% (v/v) TFA. The fractions with activities were mixed 1 : 1 with a solution of 15 mg *α*-cyano-4-hydroxycinnamic acid in 50% acetonitrile, 49.9% ethanol, and 0.1% TFA, and deposited on a ground steel Matrix-Assisted Laser Desorption Ionization target. Mass spectra were recovered in a positive reflector mode with an Ultra Flex TOF/TOF (Bruker Daltonics GmBH, Bremen, Germany) by using a pulsed ion extraction duration of 40 ns and an acceleration voltage of 25 kV.

### 2.7. Effect of Nutrient Sources and Physiological Conditions on Bacteriocin Production

The carbon sources tested were glucose, lactose, sucrose, and fructose at the concentration of 10 g·L^−1^. Cultures standardized (OD_600nm_ = 0.6) were grown in LAPT broth with glucose, lactose, sucrose, or fructose and incubated at 30°C without agitation for 24 h. Samples were examined every hour for bacterial growth (OD_600nm_), changes in culture pH, and bacteriocin production.

To study the effect of different nitrogen sources on bacteriocin production, the LAPT medium was supplemented with each different nitrogen source (tryptone, yeast extract, meat extract, animal peptone, soy peptone, and casein peptone at 35 g·L^−1^). Influence of the pH of the culture medium on bacteriocin production was determined by adjusting the initial pH of the LAPT broth to pH 4.5, 5.0, 5.5, 6.0, 6.5, and 7.0 with sterile 1 N HCl. The effect of temperature on bacteriocin production was determined by growing bacteriocinogenic cultures in LAPT medium at temperatures of 20, 25, 30, 35, and 40°C for 24 h. Bacteriocinogenic cultures were also grown in LAPT medium under anaerobic (with sealed anaerobic tubes) and aerobic conditions (with agitation). All tested broths were inoculated with standardized (OD_600nm_ = 0.6) bacteriocinogenic cultures for 24 h. After the incubation, the final pH, bacteriocin production, and bacterial growth (OD at 600 nm) were determined for all conditions described above.

### 2.8. Statistics

All experiments with regard to bacteriocin production were carried out in triplicate and repeated twice. When error bars were given in the figures, they refer to the standard deviation of the mean.

## 3. Results and Discussion

In this study, we report the identification and production of bacteriocins produced by LAB isolated from sausage (Italian type). BLAST analysis of the partial sequence of 16S rRNA gene (approximately 1500 nucleotides) of each isolate showed 99% nucleotide identity to the 16S rDNA sequence of *L. lactis* CV56, *L. lactis* SL3, and *L. lactis* KLDS (GenBank: CP002365.1, AY675242.1, and DQ340068.1) (data not shown), indicating that the bacteriocin-producing isolates are *L. lactis*.

**Figure 1:**
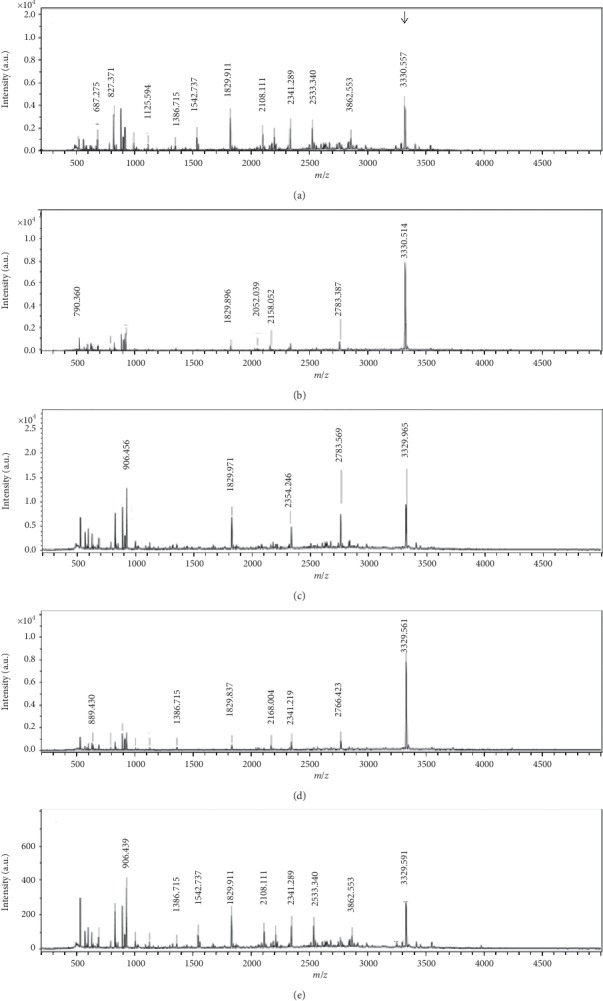
Mass spectrometry analysis of nisin Z purified from *Lactococcus lactis* ID1.5, ID3.1, ID8.5, PR3.1, and PD4.7. The arrow indicates the molecular mass of the nisin Z.

In order to purify the bacteriocins produced and secreted by five *L. lactis* strains, three steps of purification were carried out from the cell free supernatant. The highest active fraction of the bacteriocin was eluted with 30% to 40% isopropanol for all strains (data not shown). The molecular masses of the purified fractions from *L. lactis* ID1.5, ID3.1, ID8.5, PD4.7, and PR3.1 were 3330.567 Da, 3330.514 Da, 3329.985 Da, 3329.561 Da, and 3329.591 Da, respectively (Figure 1), which are similar to the molecular mass of the nisin Z (3330. 93 Da) [32].

**Figure 2:**
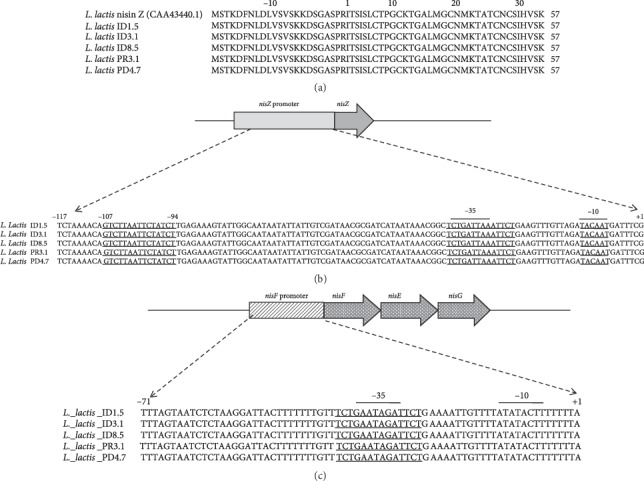
(a) Deduced amino acid sequences of the region encoding nisin Z in *Lactococcus lactis* ID1.5, ID3.1, ID8.5, PR3.1, PD4.7, and homologous sequence of *L. lactis* NIZO 22186 obtained from the GenBank. (b) Alignment of the *nis*Z promoter sequences of *L. lactis* ID1.5, ID3.1, ID8.5, PR3.1, and PD4.7. The −35 and −10 sites and TCT-N_8_-TCT are underlined. (c) Alignment of the *nisF* promoter sequences of *Lactococcus lactis* ID1.5, ID3.1, ID8.5, PR3.1, and PD4.7. The −35 and −10 sites and TCT-N_8_-TCT are underlined.

The PCR products obtained from the amplifications of genomic DNA of the five *L. lactis* strains with primers specific to nisin structural gene were subjected to nucleotide sequencing. Results indicated that the nisin gene sequences from the five strains are identical to nisin Z gene (GenBank: AB375441) (Figure 2(a)), which has an asparagine residue at position 27 instead of histidine found in nisin A [12]. Preliminary tests indicated differences in the inhibitory activity among the isolates [27]; however, these differences were not accounted for by the gene structure. Nisin Z is a bacteriocin potentially active against pathogenic Gram-positive bacteria as well as Gram-negative bacteria [33, 34]. In addition, De Vos et al. [21] reported that His27Asn substitution resulted in a higher diffusion rate of nisin Z, which in turn contributed to the larger inhibition zones produced by nisin Z in agar diffusion assays.

Analysis of the DNA sequence of the promoter obtained from genomic DNA of five *L. lactis* strains confirmed that all the promoters of the structural nisin genes were 100% identical to the published sequences of the *nisZ* and *nisA* promoters (GenBank: Y13384.1 and Z18947.1) (Figure 2(b)). The promoter sequences of *nisZ* gene of the five strains contain a partially conserved region, the TCT-N_8_-TCT motif, present at position −39 to −26, upstream of the transcription start site of *nisZ* (Figure 2(b)), which could be involved in the transcriptional control function. Chandrapati and O'Sullivan [35] reported that this motif may be involved in a cooperative binding of the NisR response regulator of the NisRK two-component regulatory system.

They also reported a second TCT-N_8_-TCT motif present at position −107 to −94, which was also confirmed in this work (Figure 2(c)). This TCT repeat, together with the first one, is involved in the optimal binding of NisR and also in the induction by some component of the Leloir pathway of galactose metabolism [36]. Thus, it has been reported that galactose and lactose can induce transcription from the *nisA* promoter [35, 36]. Another promoter in front of the *nisFEG* genes was identified in the five strains; it is identical to nisin A regulon (GenBank: HM219853) (Figure 2(c)). The sequence upstream of the *nisF* transcription start site included a single TCT direct repeat with an 8 bp spacer region similar to the TCT-N_8_-TCT motif present at the same position, −39 to −26, upstream of the transcription start site of *nisZ* (Figures 2(b) and 2(c)). The *nisZ* and *nisF* promoter sequences obtained from all strains are identical (Figures 2(b) and 2(c)).

Plasmids were found in the five strains of *L. lactis* (data not shown). It was observed that for all strains cured derivatives were able to produce bacteriocin, suggesting that the genes encoding the bacteriocin are located on the chromosome. Various researchers have found that nisin genes are present on a number of plasmids or also on the chromosome [6, 22, 37, 38]. In addition, nisin gene cluster has been shown to be located on conjugative large transposons (∼70 kb) [38], which also contain the genetic determinants of sucrose metabolism. Interestingly, it has been suggested that a genetic regulatory system or a common metabolic control system is responsible for sucrose fermentation and nisin production capacity [19, 38].

**Figure 3:**
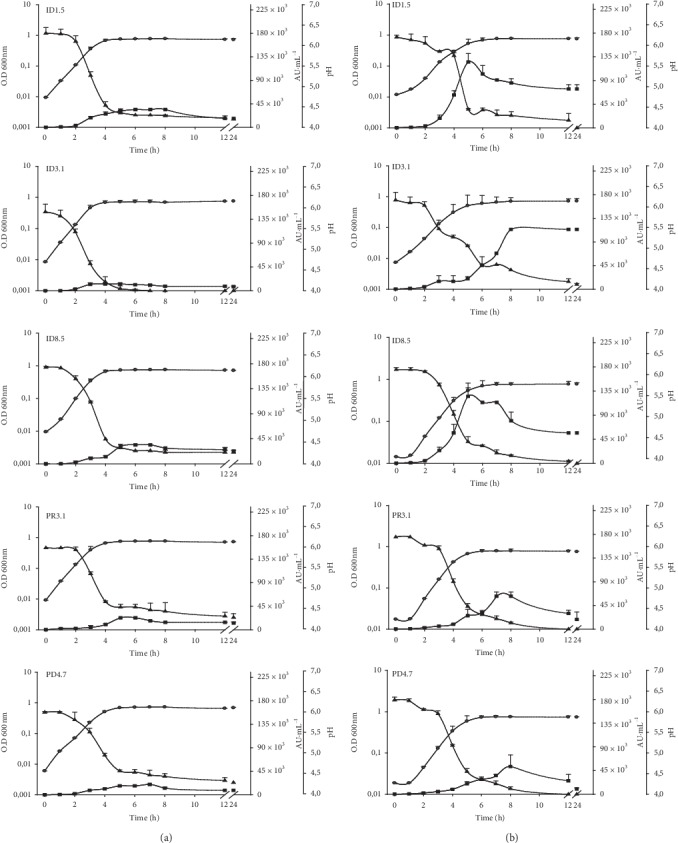
Production of nisin Z by *Lactococcus lactis* ID1.5, ID3.1, ID8.5, PR3.1, and PD4.7 strains. The left column (a) shows the results of using 10 g glucose L^−1^ and, the right column (b) shows the results of using 10 g fructose L^−1^ as carbohydrate source. Bacteriocin activity production is presented as AU·mL^−1^ (■); optical density at 630 nm (●) and changes in pH values (▲) are indicated. Each point represents the mean ± standard error of two independent experiments.

**Figure 4:**
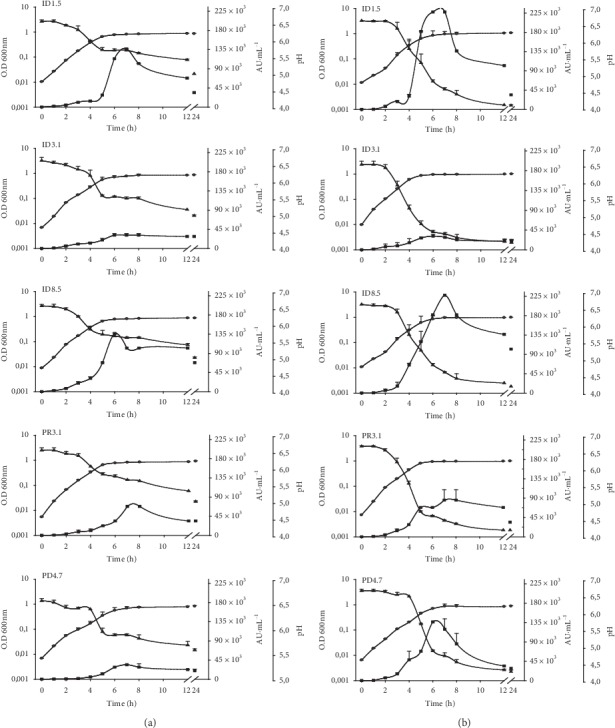
Production of nisin Z by *Lactococcus lactis* ID1.5, ID3.1, ID8.5, PR3.1, and PD4.7 strains. The left column (a) shows the results of using 10 g lactose L^−1^ and the right column (b) shows the results of using 10 g sucrose L^−1^ as carbohydrate source. Antimicrobial activity is presented as AU·mL^−1^ (■); optical density at 630 nm (●) and changes in pH values (▲) are indicated. Each point represents the mean ± standard error of two independent experiments.

The kinetics of microbial growth and nisin production of the five *L. lactis* isolates are presented in Figures 3 and 4. The bacteriocin activities increased concomitantly with an increase in the growth and reached its maximal activity at the stationary phase (Figures 3 and 4). High level of nisin production was obtained by using fructose, lactose, and sucrose as carbon sources (Figures 3(b) and 4(a)). In general, the isolate ID 3.1 appeared to produce less bacteriocin in the presence of these sugars except for fructose. Sucrose seemed to be the sugar that gave the highest bacteriocin activity in four of the isolates (but not ID3.1). For all isolates, glucose yielded the smallest amount of bacteriocin activity compared to the other sugars (Figures 3 and 4). A decrease in nisin production was observed after 6–8 h of incubation for all strains; however, it remained constant when *L. lactis* ID 3.1 was supplemented with fructose (Figure 3(b)).

In general, the isolates exhibited apparently different bacteriocin activities. The two isolates *L. lactis* ID1.5 and ID8.5 displayed strong antimicrobial activity (Figures 3 and 4). Carbon source selection has been reported as a critical control step in nisin production because of its effects on the cell growth and nisin biosynthesis [19]. For example, sucrose and lactose were determined to be efficient carbon sources for nisin production in *L. lactis* NIZO 22186 [19], *L. lactis* ATCC 11454 [39], and *L. lactis* A164 [18], while fructose was the most efficient carbon source in *L. lactis* LL27 [40] and glucose in *L. lactis* IO-1 [11]. Interestingly, poor production of nisin Z was observed in the presence of glucose. Taken together, these results show the importance of testing different carbohydrates in order to increase nisin production.

The five *L. lactis* isolates produced nisin Z efficiently from tryptone and casein peptone as sole nitrogen sources; however, the growth was similar in media supplemented with other nitrogen sources. Previous work by Simsek et al. [40] indicated that yeast extract and meat extract were the most efficient nitrogen sources for nisin A production by *L. lactis* LL27. However, our results indicated that yeast and meat extracts result in the lowest bacteriocin activity among the tested nitrogen sources. Thus, nisin Z production by the five *L. lactis* strains is stimulated by tryptone and casein peptone, but not by yeast and meat extracts.

Our results demonstrated that nisin Z production by the five *L. lactis* strains is affected by temperature, pH, and aerobic and anaerobic conditions (Table 1). All *L. lactis* strains were able to grow and produce bacteriocin at initial pH values ranging from 4.5 to 7.0, and at temperatures from 30°C to 40°C (Table 1). However, high nisin Z production was obtained when these strains were cultivated at initial pH 6.0 and 6.5, and incubated at 25°C and 30°C (Table 1). High growth rate was reached when all strains were cultivated at pH 7.0 and incubated at 35°C, but bacteriocin production was reduced under these conditions (Table 1). In contrast, Matsusak et al. [11] reported higher nisin production by *L. lactis* IO-1 at pH values ranging from 5.0 to 5.5.

Our results also showed that high temperature (40°C) did not influence the growth of the five nisin-producing strains, but resulted in reduced nisin Z production. Temperature can affect the stability of the peptide by interfering in posttranslational modification, adsorption to cells, and proteolysis of the bacteriocin [41]. In addition, temperature has an important regulatory effect on its biosynthesis. One example of a temperature-sensitive bacteriocin biosynthesis was published by Diep et al. [32], in which the biosynthesis of sakacin A occurred at 25°C and 30°C, but not at higher temperatures (33.5–35.0°C). The temperature regulation of sakacin A biosynthesis occurred at the transcription level, and the reduced bacteriocin production at high temperatures was related to a reduced synthesis of the inducer peptide. Additionally, it is known that high temperature enhances the genetic instability of the plasmid carrying the bacteriocin genes [42, 43]. However, the loss of nisin Z production by the five *L. lactis* isolates at 35°C and 40°C was not due to their genetic determinant instability because plasmid curing results suggest that the genes encoding nisin Z in these five strains are located on the chromosome.

As demonstrated in this work, the structural gene of nisin Z was identified in the five strains, which produced nisin Z at variable mounts. We analyzed *nisF* and *nisZ* promoter sequences because these promoters are controlled in the same manner; however, the sequences are similar in all strains, demonstrating that there should be other factors responsible for differential production of nisin Z.

## 4. Conclusion

In conclusion, the structural genes and molecular masses of the bacteriocins produced by five *L. lactis* strains are identical to those of nisin Z, indicating that the bacteriocin produced by each of these strains is nisin Z. Maximum nisin Z production in batch culture was achieved by two isolates *L. lactis* ID1.5 and ID8.5 (not ID3.1), at initial pH of 6.0 and 6.5, at incubation temperatures of 25°C and 30°C, under aerobic condition, and when sucrose was used as a sole carbon source. Supplementation of LAPT broth with tryptone and casein peptone increased the production of nisin Z by the five *L. lactis* strains. These parameters are important for the optimization of nisin Z production, which is essential for the use of these strains or their bacteriocins as biopreservation agents.

To our knowledge, this is the first report of nisin Z production by *L. lactis* isolated from fermented sausage in Brazil. Our study is also the first study that showed the production of identical bacteriocins at different levels and under diverse conditions, by different wild strains of *L. lactis* isolated from same environment. We suggest that identification of autochthonous strains producing higher amounts of the antimicrobials would lead to their application as starters in preservation of foods and may further help to reinforce originality of traditional foods.

## Figures and Tables

**Table 1:** Effect of nitrogen source, initial pH, temperature, and aerobic and anaerobic conditions on the production of nisin Z by *Lactococcus lactis* strains ID1.5, ID3.1, ID8.5, PR3.1, and PD4.7 strains.

Conditions tested	Antimicrobial activity (AU·mL^−1^)^*∗*^				
	ID1.5	ID3.1	ID8.5	PR3.1	PD4.7
Tryptone	125000	114000	91000	74000	57000
Animal peptone	57000	62000	51000	49000	40000
Soy peptone	23000	34000	14000	30000	34000
Casein peptone	91000	137000	57000	125000	114000
Meat extract	8600	9200	4300	5700	4300
Yeast extract	12800	12800	9300	11000	4300
Initial pH					
4.5	6400	12800	10000	12000	4200
5.0	31000	26000	20000	23000	13000
5.5	34000	32000	46000	40000	52000
6.0	80000	114000	114000	137000	137000
6.5	91000	91000	114000	114000	80000
7.0	13000	20000	23000	16000	17000
Temperature (°C)					
20	7000	6000	5000	7000	4000
25	14000	17000	17000	13000	10000
30	14000	17000	19000	14000	10000
35	6400	10000	7000	9000	3000
40	400	300	300	400	300
Aerobiose	46000	31000	51000	46000	40000
Anaerobiose	31000	17000	14000	13000	11000

^*∗*^All experiments were performed in triplicate, and the averages of two independent tests were calculated.

## Data Availability

The data used to support the findings of this study are available from the corresponding author upon request.
